# A case report of pre-eclampsia-like endothelial injury in the kidney of an 85-year-old man treated with ibrutinib

**DOI:** 10.1186/s12882-022-02873-w

**Published:** 2022-07-23

**Authors:** Amy Li, Sophia L. Ambruso, Ozgur Akin Oto, Marc Barry, Charles L. Edelstein

**Affiliations:** 1 Division of Renal Diseases and Hypertension, University of Colorado Anschutz Medical Campus, 12700 East, 19th Ave, Box C281, Aurora, CO 80045 USA; 2 Renal Section, Rocky Mountain Regional VA Medical Center, Aurora, CO USA; 3 Department of Pathology, University of Utah, Salt Lake City, UT USA

**Keywords:** Endothelial injury, Ibrutinib, Acute kidney injury, Hypertension, Endothelial swelling, Pre-eclampsia

## Abstract

**Background:**

Glomerular endotheliosis is the pathognomonic glomerular lesion in pre-eclampsia that has also been described in those taking tyrosine kinase inhibitors for cancer treatment. Ibrutinib is a Bruton’s tyrosine kinase inhibitor used to treat chronic lymphocytic leukemia (CLL). We report the first known case of glomerular endotheliosis on kidney biopsy in a patient on ibrutinib monotherapy.

**Case presentation:**

The patient presented with acute on chronic kidney disease, proteinuria, low C3 and C4 and a high rheumatoid factor titer. A kidney biopsy was performed to confirm a preliminary diagnosis of membranoproliferative glomerulonephritis (MPGN), the most common glomerular disease in patients with CLL. Unexpectedly, the kidney biopsy showed pre-eclampsia-like lesions on light and electron microscopy: occlusion of glomerular peripheral capillary lumens by swollen reactive endothelial cells. Findings of glomerulonephritis were not seen, and there were no specific glomerular immune deposits by immunofluorescence or electron microscopy.

**Conclusions:**

CLL is known to cause glomerular lesions, mainly MPGN. There is increasing evidence that ibrutinib, a major treatment for CLL, can cause kidney disease, but the precise pathology is not characterized. We present a patient with CLL on ibrutinib with signs of glomerular endotheliosis. Based on the absence of CLL-induced kidney pathologies typically seen on the kidney biopsy and the non-selectivity of ibrutinib, we attributed the glomerular endotheliosis to ibrutinib. In pre-eclampsia, increased soluble fms-like tyrosine kinase 1 (sFlt1) levels induce endothelial dysfunction by decreasing vascular endothelial growth factor (VEGF). Ibrutinib has been demonstrated to have non-selective tyrosine kinase inhibition, including inhibition of VEGF receptor (VEGFR) and epidermal growth factor receptor (EGFR). VEGFR and EGFR inhibitors have recently been described in the literature to cause hypertension, proteinuria, and glomerular endotheliosis. Kidney biopsy should be performed in CLL patients on ibrutinib that present with acute kidney injury (AKI) or proteinuria to determine whether the clinical picture is attributable to the disease itself or a complication of the therapy.

## Background

Chronic lymphocytic leukemia (CLL) is the most common chronic leukemia in adults in the United States and Europe [1]. Despite the relative frequency of CLL, renal complications of CLL are uncommon. Membranoproliferative glomerulonephritis (MPGN) is the most common renal complication of CLL. In those with MPGN, cryoglobulinemia is found in nearly 50% of cases; however, this is likely an underestimation due to the insensitive nature of the laboratory assay for cryoglobulins [2]. Other renal manifestations include membranous GN, minimal change disease, neoplastic infiltration, and amyloidosis [3–7]. Endothelial injury has not been described in patients with CLL.

Ibrutinib is an orally bioavailable Bruton’s tyrosine kinase (BTK) inhibitor that irreversibly inhibits BTK with subsequent impairment in cell proliferation, migration, and NF-kB activation that was FDA-approved for CLL in 2014 [8–10]. However, it is not specific to BTK and has been shown to bind to other kinases including but not limited to interleukin-2-inducible T-cell kinase [11], vascular endothelial growth factor receptor (VEGFR) [12, 13] and epidermal growth factor receptor (EGFR) [11, 14], which lends to ibrutinib’s profile of adverse effects that can develop even years after initiating therapy [12, 15]. The most common and/or serious side effects of ibrutinib include atrial fibrillation, ventricular arrhythmias, bleeding, infection, hypertension, heart failure, diarrhea, arthralgias, and hair/skin/nail changes [15–18]. 

Rare case reports of ibrutinib-induced renal pathology have only been recently described, including acute interstitial nephritis [19], tumor lysis syndrome [20], and acute tubular injury [21]. Additionally, in an observational study of mantle cell lymphoma patients treated with ibrutinib monotherapy, 35% of patients had increases in serum creatinine from their baseline (above the upper limit of normal) and 7% of patients had renal failure with majority of cases occurring in those with pre-existing hypertension and/or chronic kidney disease (CKD) [22]. The cause of acute kidney injury (AKI) or CKD in patients treated with ibrutinib remains largely unknown. Ibrutinib has not been reported to cause endothelial injury.

We present a case of severe, pre-eclampsia-like endothelial injury in a kidney of a CLL patient treated with ibrutinib.

## Case presentation

An 85-year-old man presented to the hospital with progressive dyspnea on exertion associated with peripheral edema for one week prior to admission. He has a longstanding history of CLL, in remission, that has been treated with ibrutinib for five years. Approximately one year prior to this presentation, he was admitted to an outside hospital for new diagnoses of severe hypertension complicated by heart failure with preserved ejection fraction. At that time, he was also found to have AKI with creatinine of 3.4 mg/dL (from prior baseline of 1.4–1.5 mg/dL). Notably, his urinalysis at that time revealed new proteinuria (3 + on dipstick urinalysis) that was not otherwise quantified, occasional granular casts, and 50–100 red blood cells. At the time, he was thought to have acute tubular necrosis (ATN) due to heart failure and hypertensive emergency as his creatinine eventually improved to 1.8–2.1 mg/dL with treatment of heart failure including diuresis and blood pressure control. The hematuria was thought to be due to ATN and there was no suspicion of a glomerular disease. On follow up after discharge, since his creatinine was still elevated from his prior baseline, he was felt to have CKD from prior NSAID use, hypertension, and heart failure. It was not recognized that his presentation at the time could be related to the ibrutinib, so the ibrutinib was continued, despite a well-documented list of adverse reactions that include heart failure and hypertension. In hindsight, it is possible that ibrutinib contributed to the clinical picture of AKI at that time. He had also not been seen by nephrology. Other past medical history included ankylosing spondylitis and hypothyroidism. Outpatient medications included amlodipine, carvedilol, hydralazine, ibrutinib, levothyroxine, and omeprazole. He has a remote history of using NSAIDs (indomethacin) for his ankylosing spondylitis but had not used any NSAIDs in over 20 years. He had a remote history of smoking but otherwise denied any alcohol or other substance use.

Clinical findings on the current admission included the following: blood pressure 186/72 mmHg, heart rate 60/min, afebrile, oxygen saturation 94% on room air; significant lower extremity peripheral edema but clear lungs on auscultation. He did have small impetiginous peri-oral skin lesions that were healing after a brief course of cephalexin. He did not have any rash, synovitis, or arthritis on examination. Laboratory workup revealed chronic normocytic anemia (hemoglobin 9.7 g/dL) with chronic thrombocytopenia (platelets 73,000/µL) that were at baseline and consistent with his history of CLL. The serum creatinine was 2.8 mg/dL (eGFR 23 mL/min/1.73m^2^), from most recent baseline of 2.1–2.4 mg/dL. Albumin was 2.8 g/dL with otherwise normal transaminases. LDL cholesterol was 74 mg/dL. He had 2.8 g of proteinuria with 1.014 g of creatinine on 24-h urine collection. Urine microscopy revealed many non-dysmorphic RBCs. Renal ultrasound revealed 11 cm kidneys bilaterally with normal echogenicity. C3 was 8 mg/dL and C4 was 40 mg/dL and rheumatoid factor was 1280. Serum protein electrophoresis revealed faint IgM kappa monoclonal gammopathy with a serum kappa-to-lambda ratio of 2. Otherwise, anti-nuclear antibodies, anti-double-stranded DNA, anti-histone antibodies, anti-neutrophil cytoplasmic antibodies, anti-glomerular basement membrane antibodies, hepatitis B and C serologies, HIV, and syphilis serologies were negative and hemoglobin A1c was 5.5%. A renal biopsy was pursued due to high clinical suspicion for MPGN given low complements and high rheumatoid factor titers that was felt to represent cryoglobulinemia, which has been described in CLL [2, 23, 24]. Other considerations in the differential prior to biopsy included lymphoid invasion of the renal parenchyma, amyloidosis [3, 4] or light-chain nephropathy [25].

The patient was treated with oral antihypertensives including carvedilol, amlodipine, and intravenous furosemide to achieve goal blood pressure < 140/90 mmHg in preparation for kidney biopsy and for volume management. Ibrutinib was discontinued upon admission in preparation for anticipated biopsy.

### Renal biopsy

**Fig. 1:**
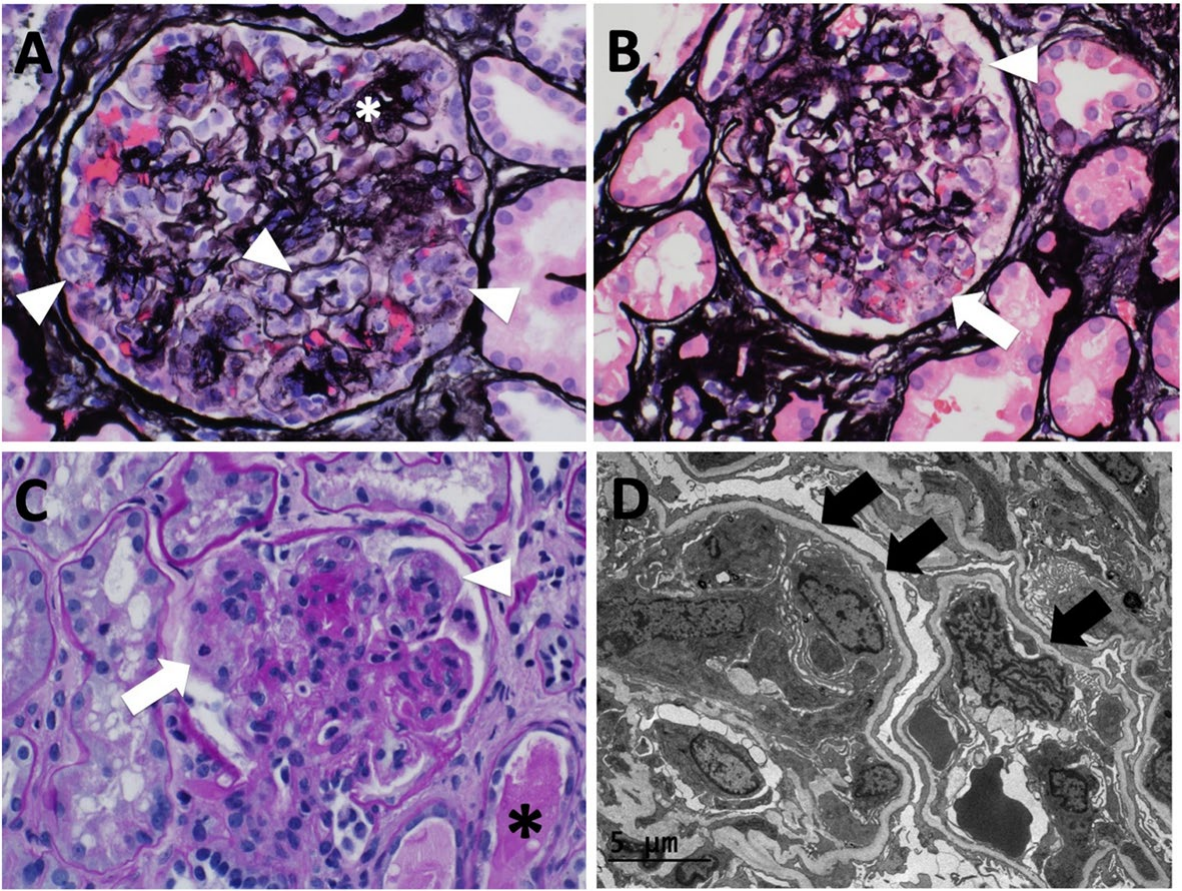
**A**-**C** Light microscopy of renal biopsy: Jones Silver (**A** and **B**) and Periodic Acid-Schiff (**C**) staining shows prominence of glomerular peripheral capillary loop cells (arrowheads), predominantly representing swollen endothelial cells. There are segmental areas suggestive for mesangiolysis (**B** and **C**: arrows). There is a background of a mild nodular increase in mesangial matrix (**A**: white asterisk). Peripheral capillary loop glomerular basement membrane double contours are not a prominent finding. There are no peripheral capillary loop thrombi or hyaline pseudothrombi. Focal acute tubular injury is seen (**C**: black asterisk) (400X magnification). Equipment used: Microscope-Olympus BX53; objective lenses – Olympus U PlanFL N; camera – Olympus DP73; Acquisition software- Olympus cellSens Standard. **D** Electron microscopy of renal biopsy: glomerular peripheral capillary loops show swollen reactive endothelial cells (arrows) and associated narrowing of capillary lumens. Overlying podocytes show effacement of foot process (arrows). Glomerular basement reduplication and cellular interposition is not seen, and there are no specific granular glomerular electron dense deposits (800X magnification)

Interventional radiology-guided left renal biopsy was performed on hospital day 7 after adequate blood pressure control. By light microscopy of the renal biopsy, glomeruli showed a prominence of peripheral capillary loop cells, comprised predominantly of swollen endothelial cells (Fig. 1A-C). Occasional possible focal segmental areas of mesangiolysis were seen (Fig. 1B-C). Glomerular basement membrane double contours were rare, suggesting recent onset of endothelial injury. No definite microthrombi or hyaline pseudothrombi seen. There was no tuft necrosis or crescent formation. Glomeruli showed a background of a mild nodular increase in mesangial matrix (Fig. 1A). Tubules showed signs of acute injury. There was also no evidence of neoplastic lymphoid infiltration of kidney parenchyma. There was approximately 20–25% interstitial fibrosis and tubular atrophy. Arteries showed moderate-to-severe sclerosis, and there was focal arteriolar sclerosis and hyalinosis. Immunofluorescence microscopy showed trace to 1 + pattern of peripheral capillary loop staining for IgM, C3, and kappa light chains, which was interpreted as being consistent with non-specific trapping and deposition of circulating factors in setting of endothelial injury. Electron microscopy confirmed the presence of glomerular peripheral capillary loop endothelial cell swelling and reactive changes and showed associated podocyte foot process effacement (Fig. 1D). There were no significant glomerular electron dense deposits and areas of peripheral capillary loop cellular interposition were a relatively rare finding.

### Clinical course

Interestingly, the renal biopsy was not consistent with any of the diagnoses considered prior to biopsy. Instead, the biopsy findings were akin to glomerular endotheliosis described in pre-eclampsia. Given his recent diagnosis of hypertension and diastolic heart failure, which are known adverse reactions to ibrutinib, we postulated that findings of the renal biopsy were most likely a consequence of ibrutinib.

Serum complements and rheumatoid factor normalized on hospital day 10. Serum creatinine peaked at 5.2 mg/dL on hospital day 11. He did not require renal replacement therapy during his hospitalization. He was discharged on hospital day 14 with serum creatinine of 4.2 mg/dL. On follow up 5 days after discharge, serum creatinine improved to 3.3 mg/dL with cystatin C level of 2.44 mg/L. In the months after discharge, the serum creatinine stabilized at 2.5 mg/dL. He was started on lisinopril for proteinuria and proteinuria improved to 0.64 g/g. Ibrutinib was discontinued indefinitely. Hematology/oncology considered other agents, such as acalabrutinib or rituximab, but treatment was deferred as the CLL remained in remission.

## Discussion and conclusions

This patient’s presentation was complex with several findings that demanded explanation, including AKI on CKD, high rheumatoid factor titers and hypocomplementemia.

CLL is well-known to produce IgM antibodies with reactivity to self-antigens, including those with rheumatoid factor activity [26–30]. Immune defects in CLL are well-described even in the absence of treatment, including hypogammaglobulinemia, cellular defects, and complement deficiencies of both the classical and alternate pathways [31]. Early studies have demonstrated complement deficiencies in CLL, including deficiencies in C1, C2, C3, and C4 [32–34] with one study demonstrating that approximately 38% of CLL patients have at least one complement deficiency [35].

Hypertension is a well-documented adverse reaction to ibrutinib that can appear even over a year after initiating therapy [15–17]. Our patient had no prior history of hypertension until one year prior to presentation (at his first admission). At that time, he had been on ibrutinib for four years. Blood pressure had been challenging to control despite using multiple agents such as carvedilol, hydralazine, amlodipine, and furosemide (the latter was intermittently used as outpatient). As such, hypertension is the most likely explanation for the mesangial nodularity, arterial sclerosis, and focal arteriolar sclerosis and hyalinosis, particularly since the patient does not have a history of diabetes mellitus.

The marked endothelial injury and swelling observed on the patient’s renal biopsy associated with ibrutinib is a novel finding that has not been previously reported in the literature. Our patient presented with hypertension, proteinuria, and endothelial injury mimicking that of endotheliosis, which are all hallmarks of pre-eclampsia. Endotheliosis is thought to be responsible for the decreased eGFR in pre-eclampsia via reduced ultrafiltration coefficient as opposed to decreased renal blood flow [36]. In pre-eclampsia, a non-specific pattern of immunofluorescence can be observed due to non-immunologic insudation. This occurs in the setting of endothelial injury, which allows circulating factors to deposit in the glomerulus, which is often confirmed by the absence of electron-dense deposits on electron microscopy. This pattern was similar to that observed in this patient.

The mechanism of the ibrutinib-induced endotheliosis is best explained by ibrutinib’s non-selective inhibition of VEGF. Under physiologic conditions, VEGF is an important factor in maintaining endothelial integrity of the vascular beds, which has been demonstrated in the kidney and liver [37, 38]. VEGF is also one of the most well-studied angiogenic factors in cancers, including CLL, which has been shown to be sensitive to VEGF [39]. Clinically, there are no VEGF-specific inhibitors approved for treatment of CLL.

In pre-eclampsia, circulating soluble fms-like tyrosine kinase 1 (sFlt1 or VEGFR-1), is upregulated. Increased sFlt1 levels induce endothelial dysfunction by decreasing VEGF, particularly in fenestrated endothelial cells with constitutive expression of VEGF, such as those found in the glomerulus [38]. VEGF-specific inhibitors, such as bevacizumab and apatinib, have been well-described in the literature to cause proteinuria, hypertension, renal failure, and less commonly, thrombotic microangiopathy and acute tubular necrosis [40, 41]. Bevacizumab, specifically, has been reported to cause endothelial swelling with thrombotic microangiopathy and podocyte foot process effacement [40]. Furthermore, sunitinib and sorafenib are also non-selective tyrosine kinase inhibitors that have been described to cause pre-eclampsia-like syndrome [42]. Sunitinib most potently inhibits VEGFR, platelet-derived growth factor (PDGF), and c-Kit. Sorafenib inhibits multiple intracellular and cell surface kinases, including but not limited to VEGFR and PDGF receptor. Although ibrutinib has not clinically been observed to cause a similar pattern of injury prior to this case, BTK inhibition has been shown to suppress production of VEGF by macrophages [13]. Furthermore, there is evidence that ibrutinib is a non-selective inhibitor of the VEGFR [12].

Another molecular mechanism that potentially contributed to the pattern of endotheliosis seen in our patient includes epidermal growth factor receptor (EGFR) inhibition by ibrutinib. Due to some non-selectivity, ibrutinib has demonstrated EGFR inhibition [11, 14]. To support this notion, erlotinib and gefitinib, EGFR tyrosine kinase inhibitors known to treat non-small cell lung cancers, have recently been described in the literature to cause hypertension, proteinuria, and glomerular endotheliosis [43]. EGFR is highly expressed in the adult human kidney, particularly in the glomerular mesangial cells. EGFR is known to play a key role in renal organogenesis, intra-renal hemodynamics, and electrolyte homeostasis [44, 45]. EGF induces constriction of pre- and post-glomerular arterioles, which produces physiologically significant reduction in glomerular filtration and renal blood flow.

Clinically, when a VEGFR inhibitor (such as ramucirumab) is administrated with an EGFR inhibitor (such as erlotinib), the incidence of proteinuria increased greatly compared to VEGFR inhibitor alone [46]. This further supports the hypothesized mechanism that the non-selective inhibition of VEGFR and EGFR (in addition to BTK) by ibrutinib causes endothelial injury, despite that this type of renal injury had never been formally described in the literature.

Next, the question arose as to why proteinuria and podocyte foot process effacement developed with ibrutinib. There are multiple molecular pathways implicated in disrupting the endothelial-podocyte microenvironment, precipitating proteinuria, podocyte injury, and endothelial injury [47], two of which notably include VEGF and EGFR. Podocytes produce VEGF and the VEGFR is expressed by glomerular endothelial cells. It was demonstrated in a podocyte-specific VEGF-knockout murine model, that loss of podocyte-derived VEGF precipitates microvascular injury, including thrombotic microangiopathy, followed by systemic hypertension [40]. This finding led to the predominant hypothesis that VEGF production from podocytes is critical for promotion of endothelial fenestrae and overall appropriate maintenance of the endothelium. It also further suggests that hypertension may not be the initial trigger for the endothelial injury and thrombotic microangiopathy.

In conclusion, we presented a complex case of pre-eclampsia-like endothelial injury akin to endotheliosis in a patient on ibrutinib. To our knowledge, this is the first case of endotheliosis associated with ibrutinib. Overall, we strongly suspect that the ibrutinib was the cause of the abnormal acute findings on kidney biopsy. While renal adverse reactions to ibrutinib are rare, it may be that they are underreported in the literature as many other tyrosine kinase inhibitors have documented adverse reactions such as acute kidney injury, pre-eclampsia-like lesions, proteinuria, and thrombotic microangiopathy. The mechanism of endothelial injury caused by ibrutinib is most likely driven by non-selective inhibition of VEGF predominantly but also EGF, which are two factors with critical function to maintain the endothelial-podocyte microenvironment in the kidney. Furthermore, our patient’s recent diagnoses of hypertension and diastolic heart failure are also aligned with known cardiovascular toxicities of ibrutinib via VEGF inhibition. The precise natural history and expected rate of improvement, particularly with withdrawal of the offending agent, is not known given unclear incidence and etiologies of renal injury with ibrutinib. Furthermore, it is unclear if the new generation of BTK inhibitors, such as acalabrutinib and pirtobrutinib, have a similar risk. Patients on ibrutinib and newer generation of BTK inhibitors should be closely monitored for cardiovascular and renal complications, including kidney injury. A renal biopsy should be performed in CLL patients on ibrutinib that develop AKI and/or proteinuria as pathology representing the underlying hematological malignancy may require more aggressive treatment while pathology as described in this report that represents a complication of therapy may require discontinuation of chemotherapy or alternate treatment.

## Data Availability

Data sharing is not applicable to this article as no datasets were generated or analyzed during the current study.

## Abbreviations

CLL: Chronic lymphocytic leukemia
AKI: Acute kidney injury
CKD: Chronic kidney disease
MPGN: Membranoproliferative glomerulonephritis
BTK: Bruton’s tyrosine kinase
VEGFR: Vascular endothelial growth factor receptor
EGFR: Epidermal growth factor receptor
SFlt1: Soluble fms-like tyrosine kinase, VEGFR-1
PDGF: Platelet-derived growth factor
